# Non-invasive Hemoglobin Screening for Detection of Postpartum Anemia

**DOI:** 10.1089/whr.2024.0028

**Published:** 2024-07-19

**Authors:** Partha Pratim Das Mahapatra, Nirmal Kumar Mohakud, Chaitali Roy, Harshavardhan Rajagopal, Sandeep Sharma

**Affiliations:** ^1^ EzeRx Health Tech Private Limited, Bhubaneswar, India.; ^2^ Kalinga Institute of Medical Sciences, Bhubaneswar, India.; ^3^ American Hospital, Dubai, UAE.

**Keywords:** women health, hemoglobinometer, non-invasive, postpartum, EzeCheck

## Abstract

**Introduction:**

Postpartum period is a critical period for a woman, where the body of the woman is in healing stage. In this situation, there arises the risk for developing anemia, if proper diet is not maintained. So, it is necessary to routinely determine the hemoglobin levels during this period to avoid chances of developing anemia and assist in early diagnosis.

**Methodology:**

A cross-sectional study was carried out at the maternity ward of Kalinga Institute of Medical Sciences Hospital, Bhubaneswar. The study was conducted for a period of 5–6 months by received approval from the concerned institutional committee. The study involved women participants having age above 18 years and who had recently undergone delivery. Written informed consent was taken from all the participants prior to their enrolment for this study.

**Results:**

A total of 670 women were involved in this study and more than 50% of the women were found to be affected by anemia. A Pearson’s correlation of 0.87 was observed with sensitivity of 95.69% and specificity of 67.06% between the hematology analyzer and non-invasive EzeCheck device in determining anemia.

**Conclusion:**

Women often neglect their health situations and always prioritize their family’s wellbeing and health over their own. This avoidance results in the development of a chronic disease which in the long run becomes difficult to be cured. So, in such situations, it is necessary to inculcate certain routine tests for the women during their healing period (such as postpartum stage) to keep an eye on their health status. Use of non-invasive devices can help in achieving this in a painless and much effective manner with instant reporting of the results.

## Introduction

Anemia is defined as a condition wherein the body’s physiological necessities are not met because of hindered oxygen-carrying capacity of blood, occurring owing to reduced levels of hemoglobin (a protein that carries oxygen) as defined by the World Health Organization.^1^ The root cause for it is the lack of iron, which is seen in around half of the cases. About 53% of women and 23% of men in India somewhere in the range of 15 and 49 years old are anemic.^2^ In the current scenario, the situation has not improved much, and children and women continue to be highly affected by anemia as compared with men. Similarly, the condition of anemia is critical for the pregnant women.

India is known as the home for the largest number of anemic pregnant women, which adversely affects the health of both the mother and the child.^3,4^ Anemia during the pregnancy time period is linked with functional decompensations involving immune depression, increased chances of susceptibility to infections, low birth weight of the newly born, and high maternal morbidity and mortality rates.^4^ This condition is faced by 10%–40% of the women population.^5^ In pregnant women, the risk of anemia isn't confined to the pregnancy itself; it extends to the period following delivery, commonly referred to as postpartum anemia. After several studies, it has been anticipated that in India around 20% of the maternal deaths are directly linked to anemia and in another 20% cases, anemia was a causative factor for the same. Postpartum anemia can adversely increase the risk for blood transfusion, postpartum depression, impaired interactions between the mother and the child, and increased mortality rates, and has significant potential consequences for the future neurodevelopment of the newly born.^6–8^ Postpartum anemia is defined as the hemoglobin levels <11 g/dL, which is being observed within 2 weeks after the delivery.^9^ It is an important health issue globally and is known to cause high rates of maternal and perinatal deaths. Further studies have also revealed that in 50% of the cases where women had Hb <10.5 g/dL during delivery, they had developed moderate to severe postpartum anemia with decreased levels of Hb (below 9.5 g/dL).^8,10^ Also, the health of the newly born is also affected if the mother is anemic. Findings have shown that preterm birth and low birth weight rates are found to be high for those cases where the mother’s Hb levels are < 7 g/dL.^11^ The main cause for this is the blood loss of <400–500 mL during the delivery process leading to the depletion of the mother’s body iron reserves, which cannot be replenished easily only through the diet. The death rates of the women owing to postpartum anemia is much higher and is one of the most common causes of death in the developing countries.

To ensure proper health and functioning of both the mother and the newly born, it is very much essential to monitor the Hb levels not only before delivery or during pregnancy but also after 1–2 weeks of delivery and if required even after 8 weeks. Despite postpartum anemia being a major and a serious issue, often it is being neglected by us, mainly because of the lack of knowledge and owing to large medical expenses involving tests and hospital stays. But the problem needs to be eradicated for which an affordable and a convenient solution needs to be followed. For this, a non-invasive solution has been developed by EzeRx Health Tech called the EzeCheck, which is an affordable screening device that detects the Hb levels, provides the instant results, and is pain-free.

## Methodology

This prospective observational comparative study was done to assess the efficacy of a non-invasive screening device that could be used for the routine screening for postpartum anemia diagnosis and treatment of women in the maternity ward. The study was conducted at Kalinga Institute of Medical Sciences (KIMS) Hospital, Bhubaneswar, Odisha, in India from February 6, 2022 to July 31, 2022. The study was approved by the institutional ethical committee, KIMS, Bhubaneswar (Ref. No.: KIIT/KIMS/IEC/543/2021) and cases were included after their written informed consent to participate in this study.

All the procedures were performed in compliance with the relevant laws and institutional guidelines. All admitted women above 18 years of age, who were shifted to the maternity wards and cabins after the delivery and for whom venous Hb was sent for the part of the necessary hematological investigation, were enrolled in this study. The range of duration for enrolling the individuals for non-invasive testing was minimum 2 days to maximum 2 weeks after delivery. Women who had applied mehndi on the fingertip or had any skin-related disease, which affects the upper dermal layer of the skin, or denied for the consent were excluded from the study. For the demographic details, patient’s age was recorded.

The non-invasive Hb (NHb) was performed using the EzeCheck device just after the collection of the venous blood sample with a maximum allowed time difference of 30 minutes between the invasive and the non-invasive methods in all the studied cases. Whenever the laboratory Hb (LHb) testing was performed for routine examination, we ensured that the NHb testing was also performed. Even if repeated LHb testing was conducted for routine check-ups, the NHb testing was not repeated for the same individual who has been previously tested using the device within the time duration of 2 weeks. EzeCheck collects the absorption signal from the anterior side of the left-hand ring finger of the studied women with appropriate light shielding to avoid any sort of passing of the light from the finger bed area. After entering the patient’s age and gender on the mobile app and clicking on the start button, the signal collection and processing starts automatically for determining the Hb levels. After running a series of signal processing techniques at the backend, final processing outputs are analyzed by machine learning algorithm and value of hemoglobin is generated instantly at your mobile screen. Each participant was measured once, which took approximately 30–40 seconds, for generating the results.

NHb assessment was carried out by a staff nurse (trained to use EzeCheck). Venous Hb (LHb) was measured in the laboratory by a standard hematology analyzer (Sysmex XN 1000) by the laboratory technician, who was blinded to the NHb measurement. The Hb values obtained by both the methods were expressed in gm/dL unit. Postpartum anemia was confirmed if the hemoglobin level of <11.0 g/dL was observed within 48 hours of delivery or the participant had Hb level of <11.0 g/dL even at 2 week postpartum. Few patients who had complications were hospitalized till 2nd week after delivery, so the female patients who had a postpartum period of maximum 2 weeks were also enrolled in this study. Women included in this study were divided into two groups: anemic (Hb <11 g/dL) and nonanemic (Hb >11 g/dL), according to the obtained LHb results.

Descriptive statistics were summarized as number, percentage, range, and mean ± standard deviation (SD). For validation of the device, the bias was calculated as the absolute difference between NHb data and LHb data. Bland–Altman plot was used to assess agreement between the NHb and gold standard LHb. For this, the mean and the SD of the differences between the non-invasive and invasive measurements were calculated. The limit of agreement (LOA), which was equal to ±1.96 × SD of the mean difference, was also calculated, as described by Bunce, 2009.^12^ Both values were considered to be in agreement if majority of values fell within ±1.96 SDs. Sensitivity and specificity of the non-invasive device in all the three cases (overall, anemic, and nonanemic) were also calculated to evaluate the device accuracy for the postpartum cases. Pearson’s correlation coefficient (PCC) between the estimated NHb and LHb were also calculated to assess the strength of the association between Hb levels measured using the non-invasive device and that using the invasive gold standard method. Subgroup analysis of PCC and Bland−Altman plot were done for anemic versus nonanemic women. Value of *p* <0.05 was considered statistically significant and all the data analysis was done by using Python version 3.9 and Microsoft Excel.

## Results

Of the 670 women who were included in the original study, 418 (62.39%) women were found to be anemic (Hb <11 g/dL). Rates of anemia were significantly higher among women at the extremes of reproductive age (younger than 20 years and 40 years or older). The mean age of the women was 27.27 ± 5.4 years, with a minimum age of 18 and a maximum age of 45.

**FIG. 1.**
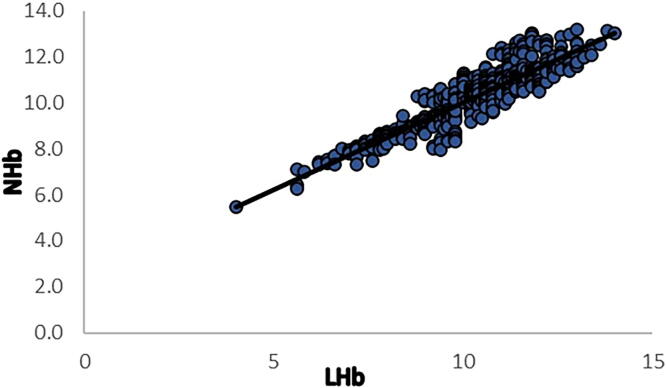
Pearson’s correlation of EzeCheck (NHb) with the hematology analyzer (LHb). LHb, laboratory Hb; NHb, non-invasive Hb.

**FIG. 2.**
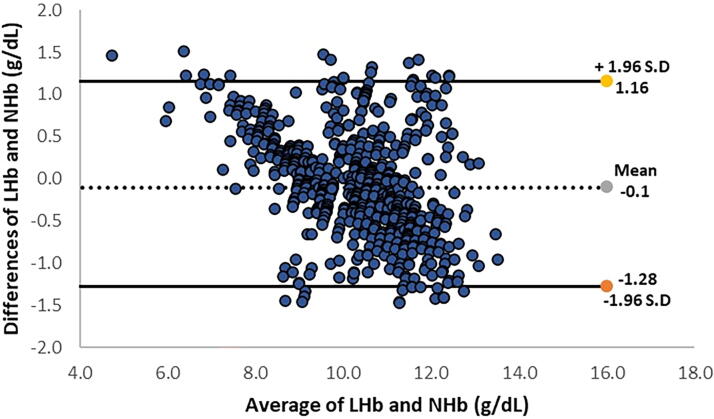
Bland–Altman plot for Hb level by using EzeCheck (NHb) and hematology analyzer (LHb).

Overall, 7.16% of the included women had hemoglobin levels <8 g/dL or severe postpartum anemia. The measured Hb levels for both the invasive method (10.33 ± 1.48 g/dL) and the non-invasive device (10.26 ± 1.23 g/dL) were found to be in close proximity. We also found a positive correlation [Pearson’s *r* = 0.911, *p* < 0.00001 between Hb levels measured using the non-invasive device and those obtained from the blood sample (Fig. 1)]. The mean difference of bias and SD of precision was −0.1 ± 0.62 g/dL (Table 2). Values of the lower and upper LOA were −1.28 and 1.16, respectively (Fig. 2). The LOAs were calculated by determining the product of bias [average of the differences between the two methods (NHb − LHb)] ± 1.96 × SD.

**FIG. 3.**
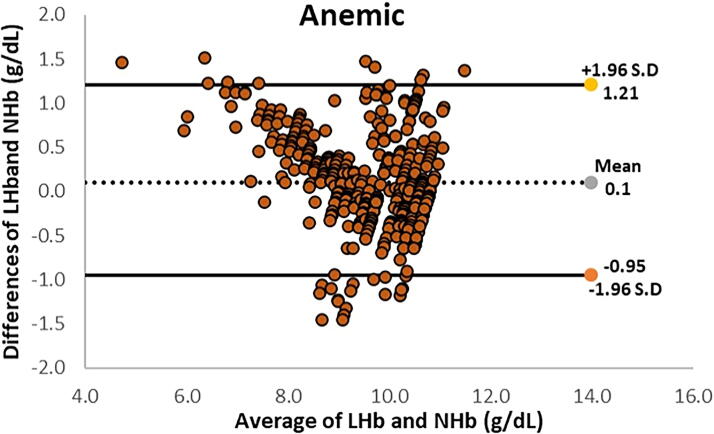
Bland–Altman plot for anemic cases by using EzeCheck (NHb) and hematology analyzer (LHb).

The overall specificity was 67.06% and sensitivity was 95.69% at 95% of allowed class interval. Statistical information of this is represented in Table 1. The descriptive statistics of the non-invasive and invasive Hb measurements are shown in Table 2 and the relationship of anemia with respect to age is shown in Table 3. For anemic cases, the LOAs were from −0.95 to 1.21 g/dL, with a mean bias of 0.1 g/dL (Fig. 3). Only 29 (6.9%) paired observations remained outside the 95% LOA.

**Table 1.** Details for Sensitivity and Specificity of EzeCheck

	NHb negative (nonanemic)	NHb positive (anemic)
LHb negative (nonanemic)	TN = 169	FP = 83
LHb positive (anemic)	FN = 18	TP = 400

FN, false negative; FP, false positive; LHb, laboratory Hb; NHb, non-invasive Hb; TN, true negative; TP, true positive.

**Table 2.** Statistical Details of the LabHb and the Non-invasive (NHb) for 670 Postpartum Cases

	Overall (*n* = 670)	Anemic (*n* = 418)	Nonanemic (*n* = 252)
Pearson’s correlation	0.911	0.877	0.567
Mean ± SD	−0.1 (0.62)	0.1 (0.55)	−0.4 (0.60)
Bias (95% CI)	−1.28 to 1.16	−0.95 to 1.21	−1.57 to 0.8

CI, confidence interval; SD, standard deviation.

**Table 3.** Relationship Between Postpartum Anemia and Age of Women (*n* = 670)

Age groups	Number “*N*” (%)	Anemic *n* = 418 (%)	Nonanemic *n* = 252 (%)
<21	57 (8.5)	35 (61.4)	22 (38.6)
21–34	542 (80.89)	327 (60.33)	215 (39.67)
34+	71 (10.6)	56 (78.87)	15 (21.13)

## Discussion

In India, several women lose their life owing to anemia either during their pregnancy or during the postpartum period. One of the major causes for this is the delayed detection of the disease. In such circumstances, the need for an effective and affordable screening method that can detect the anemia at its initial stage arises. For this, a study revealed that after the implementation of an innovative screening protocol in Israel, there was an increase of 22% in the diagnosis of moderate-severe anemia, which helped in improving the rate of anemia diagnosis, thereby reducing the delay in the treatment process, which often causes complications.^13^

In this study, we compared the hemoglobin values obtained through the laboratory along with the non-invasive EzeCheck device to determine its effectiveness in detecting anemia. We observed around 7.16% of the total studied population to be severely anemic (Hb <8 g/dL), whereas the rate of prevalence of anemia in the entire studied population was 62.4% (Hb <11 g/dL). The EzeCheck device was able to identify 400 cases to be truly positive out of 418 positive cases. Truly positive means that as per the LHb value, the patient was anemic and the NHb value also showed it as anemic. This can easily help in early detection of anemia and can be used as a preindicator to decide whether to perform the blood test or not. The predictive value for the nonanemic cases was low as compared with the anemic cases, as high false positive was observed for NHb (83 out of 252). False positive refers to the condition where the LHb value shows nonanemic but the NHb showed it as anemic. This could be possibly owing to the fact that measuring the Hb levels using the non-invasive method is not as simple as measuring the other parameters such as SpO2 non-invasively.^14^ Certain environmental factors such as inadequate finger placement, poor blood circulation, improper light coverage, and low body temperature can hinder with the NHb values and adversely affect the accuracy.

Various other non-invasive devices for Hb are also available in the market but owing to their low specificity and sensitivity levels, their use in the clinical practice is still questionable. In this study, we have not directly compared with the other available non-invasive Hb devices, but using thorough review study, it was observed that devices such as Masimo Pronto-7 Hb had had well correlation with the laboratory values in case of male patients but failed to have such good correlation in the female cases.^14^ During another comparison-based study, it was found that the Pronto-7 and NBM-200 (Orsense) devices had higher LOAs (>2 g/dL).^15^ Another device namely, HemoCue (Invasive), is now being used at several places to detect the Hb levels as it uses single drop of blood for detecting the Hb values. Although, it provides a quick and acceptable valuation of hemoglobin compared with laboratory values, it requires blood sample, trained laboratory technician, and involves use of certain chemicals and cuvettes which are expensive and sometimes unavailable for all locations.^16,17^ Also, a comparison-based study on HemoCue, Masimo Pronto, and Apple and Android cell phone applications showed that the invasive device has good correlation (0.87) with the Complete Blood Count analyzer as compared with Masimo (0.29), Apple (0.08), and Android (0.11) cell phone applications.^18^ The correlation between the LabHb and HemoCue was found to be good in case of overall population but when it was observed in case of pregnant women, the correlation was 0.58, which signifies that there is variation in its efficacy when observed in different category of the population.^19^

When comparing the sensitivity and specificity of EzeCheck (95.69% and 67.06%, respectively) with other available devices (63.9% sensitivity and 48.2% specificity for Apple, 36.1% sensitivity and 67.6% specificity for Android, 45.7% sensitivity and 85.3% specificity for Masimo Pronto, and 66.7% sensitivity and 97.6% specificity for HemoCue Hb-801)^18^, it was observed that EzeCheck demonstrates higher sensitivity than all other devices studied. However, its specificity is lower compared to Masimo and HemoCue, possible reason for which has been previously discussed. The negative predictive value for the EzeCheck device was found to be 90.37%, which is in association with the other available devices that have their overall negative predictive value >88%, whereas the positive predictive value was 82.82%, which is greater than both the invasive (80%) and the other non-invasive (15%–30%) devices.

EzeCheck could be a useful screening tool for the patient care that would allow faster detection of anemia and help in further investigation and management of the disease at its initial level, particularly for the critical patients such as females in their postpartum period. Also, by using the NHb screening, we can easily reduce the transportation costs of those patients who visit the hospitals from distant places, particularly those coming from the rural areas. Early detection of anemia in the women population could also help in reducing the risks associated with the abnormalities and complications that could arise in the newly born, thereby helping to take precautionary steps for both the mother and the neonate.

Also, the use of non-invasive devices reduces the chances of iatrogenic blood loss, which occurs owing to repeat sampling (if required).

One major limitation of our study is that we have studied the efficacy of NHb only on the postpartum women population, making the study to be centered around a single type of population. For this, the effectiveness of the non-invasive device in other categories of population needs to be studied, which could additionally support this study and help us in determining the potential of the non-invasive Hb-detecting device. Additionally, it was observed that, in case of anemic individuals, the EzeCheck device overestimated the low hemoglobin values (Hb value <8 g/dL) owing to limited data availability, which could be improved in the long run by incorporating huge data sets in the device Artificial Intelligence model. Despite this, the method was found to be quite helpful for determining the hemoglobin levels on a preliminary basis.

## Conclusion

Postpartum anemia is a severe condition which is often neglected and requires regular check on the Hb levels during the initial 2 weeks after the delivery. Regular blood tests are not possible in all cases as it is painful and is not acceptable by everyone. So, non-invasive devices such as EzeCheck could be easily used in such cases as a screening method to determine the Hb values and, if required, one can proceed for the blood test depending upon the results. As it has good correlation and narrow LOAs, it is clinically acceptable and can be used for the screening purposes.

Being a non-invasive technology, the device has certain limitations such as it cannot be used for women who have applied mehndi or have any pigmentation on the left-hand ring finger tip, as it will obstruct the light passage and can lead to erroneous values.

## Abbreviations Used

CI: Confidence Interval
Hb: Hemoglobin
KIMS: Kalinga Institute of Medical Sciences
LHb: Laboratory Hemoglobin
LOA: Limit of Agreement
NHb: Non-invasive Hemoglobin
